# The socioeconomic burden of spinal muscular atrophy in Saudi Arabia: a cross-sectional pilot study

**DOI:** 10.3389/fpubh.2024.1303475

**Published:** 2024-02-01

**Authors:** Khloud Mubark Alotaibi, Mohannad Alsuhaibani, Khalid S. Al-Essa, Ahmed Khamis Bamaga, Amnah S. Mukhtar, Ali Mohammed Alrumaih, Huda F. Al-Hasinah, Shaikhah Aldossary, Fouad Alghamdi, Mohamad-Hani Temsah, Norah Abanmy, Monira Alwhaibi, Yousif Asiri, Yazed AlRuthia

**Affiliations:** ^1^Department of Clinical Pharmacy, College of Pharmacy, King Saud University, Riyadh, Saudi Arabia; ^2^Neurology Division, Department of Pediatrics, Faculty of Medicine, King Abdulaziz University, Jeddah, Saudi Arabia; ^3^Pharmaceutical Care Department, King Faisal Specialist Hospital and Research Centre, Jeddah, Saudi Arabia; ^4^Pharmaceutical Care Department, General Directorate for Health Services, Riyadh, Saudi Arabia; ^5^Department of Pharmacy, Prince Sultan Medical City, Riyadh, Saudi Arabia; ^6^Department of Pediatric Neurology, King Fahad Specialist Hospital, Dammam, Saudi Arabia; ^7^Department of Pediatrics, College of Medicine, King Saud University, Riyadh, Saudi Arabia; ^8^Pharmacoeconomics Research Unit, College of Pharmacy, King Saud University, Riyadh, Saudi Arabia

**Keywords:** access to treatments, burden of disease, Saudi Arabia, spinal muscular atrophy, out-of-pocket (OOP) expenses

## Abstract

**Background:**

Spinal muscular atrophy (SMA) is a rare debilitating condition with a significant burden for patients and society. However, little is known about how it affects Saudi Arabia's population. The socioeconomic and medical characteristics of affected SMA patients and their caregivers are lacking.

**Purpose:**

This study aimed to describe the socioeconomic and medical characteristics of SMA patients and caregivers in Saudi Arabia.

**Patients and methods:**

A cross-sectional questionnaire-based study was conducted using snowball sampling. Assessment tools including EuroQol (EQ-5D-5L) and visual analog scale (EQ-VAS), Generalized Anxiety Disorder 7-item (GAD-7), Patient Health Questionnaire (PHQ-9), and Costs for Patients Questionnaire (CoPaQ) were used to assess the quality of life (QoL), anxiety, depression, and out-of-pocket expenditures.

**Results:**

Sixty-four caregivers of SMA patients participated. Type I patients had higher sibling concordance, ICU hospitalization, and mechanical support needs. Type III patients had better QoL. Type I patients' caregivers had higher depression scores. Type III patients' caregivers had higher out-of-pocket expenditures. Forty-eight percent received supportive care, while others received SMA approved therapies.

**Conclusion:**

SMA imposes a significant socioeconomic burden on patients and caregivers, requiring more attention from the healthcare system. Access to innovative therapies varied across SMA types. Pre-marital screening and early detection are crucial to reduce disease incidence and ensure timely treatment.

## 1 Introduction

Spinal Muscular Atrophy (SMA) is a hereditary neurodegenerative disease that primarily affects nerve cells in the anterior horn of the spinal cord, leading to irreversible degradation of α-motor neurons within the anterior horn cells and brain stem nuclei (1, 2). This genetically-linked neuromuscular condition significantly impacts the musculature of the upper limbs, reducing working capacity and ultimately causing respiratory distress due to diaphragm involvement, placing considerable burden on caregivers and decreasing the patient's life expectancy (3, 4).

Approximately 95% of SMA cases arise from homozygous deletions or mutations in survival motor neuron 1 (SMN1) on chromosome 5q13, leading to a decrease in SMN protein expression (5). However, the SMN2 gene serves as a compensatory mechanism, although only about 10–20% of SMN2-expressed protein is fully functional (1). Therefore, increased SMN protein copy numbers may alleviate disease severity (6).

SMA has diverse clinical presentations, divided into five types based on disease progression and symptom onset, with varying impacts on life expectancy (7). Furthermore, its manifestation is often categorized into four phenotypes according to motor function and age of onset (8). The Werdnig-Hoffmann variant (Type I) is the most prevalent and severe diagnosed within the first 6 months of life, is little bit less progressive and diagnosed early in the childhood (e.g., between 6 and 18 months), the Kugelberg-Welander (Type III) is the mildest and is diagnosed after the child's first 18 months, while type IV is a rare and mild type of the disease and its symptoms mostly appear in the mid-thirties (6). Symptomology often includes symmetrical muscle weakness, respiratory complications, and paralysis in severe cases (3, 9).

The management of SMA primarily focuses on supportive care, encompassing provision of sufficient nourishment, respiratory support, and mitigation of muscular weakness effects through therapeutic interventions or preventative measures (10). This includes hospitalizations necessitated by complications such as pulmonary issues, growth failure, and orthopedic problems, and common supportive therapies such as ventilators, feeding, secretion suction, and orthosis support (10–13).

As of now, the FDA has approved three gene therapies for SMA treatment: Nusinersen (Spinraza^®^ Biogen), Onasemnogene abeparvovec (Zolengesma^®^ Novartis), and Risdiplam (Evrysdi^®^ F. Hoffmann-La Roche) (10). Despite their high cost, these therapies have demonstrated effectiveness by improving patient outcomes (14).

Despite ongoing research, the exact prevalence of SMA is difficult to ascertain. However, estimates suggest that it ranges from 1 to 2 per 100,000 individuals, with an incidence of 8–10 per 100,000 live births (15). Higher prevalence rates have been observed in Middle Eastern countries such as Saudi Arabia, where recent studies estimated roughly 2,265 SMA patients, possibly due to prevalent consanguineous marriages (16).

The varying severity of SMA has significant impacts on patients and their families, leading to financial strain, psychological challenges, sleep disturbances, and social limitations (17, 18). Comprehensive support mechanisms are needed, including psychological counseling, legal advice, genetic counseling, and family planning.

The economic burden of SMA is considerable and appears to be rising. The mean annual per-patient total cost of illness (direct medical costs, non-medical costs, and informal care) from the societal perspective varies between countries ranging from $97,300 (SMA type III) to $243,930 (SMA type I) in Australia, $60,770 (SMA type III) to $124,920 (SMA type I) in Germany, and $17,790 (SMA type III) to $39,520 (SMA type I) in Italy (19). Canadian research involving over 900 patients and caregivers highlighted significant costs to families of SMA patients, with median health expenditures for assistive devices, healthcare professional services, and accommodation and travel, along with a notable negative impact on patient quality of life (20). Although SMA is a rare health condition, its incidence rate is believed to be increasing in the kingdom mainly due to high rate of consanguinity and allocating more financial resources for preventative (e.g., premarital screening) and early detection and treatment measures (e.g., newborn screening, early initiation of therapy) is necessary (16).

Details on the specific characteristics of patients affected, the financial costs of the disease, the types of treatments provided, and the emotional state of caregivers, particularly in countries such as Saudi Arabia, are scarce (8). Future research should aim to evaluate and portray a more comprehensive landscape of the burden of illness across all SMA types. This endeavor will likely provide valuable insight for future healthcare planning and support for both SMA patients and their caregivers.

Moreover, there is a scarcity of information about how the disease impacts the population of Saudi Arabia specifically. This includes, but is not limited to, the individual characteristics of affected patients, the financial implications of the disease, the types of treatments administered, and the psychological state of caregivers.

Given the significance of these aspects, a detailed cost analysis is indispensable. This would enable a comparison between the expenditures associated with future potential curative therapies and the present palliative treatments, providing a clearer financial perspective on SMA management.

It would also be insightful to determine the rates of hospitalization and usage of mechanical ventilation. These metrics could serve as indicators of the disease's severity within the affected population. Furthermore, an assessment of the emotional toll on caregivers is needed to illuminate their perceptions, expectations, and strategies while caring for family members with SMA.

For the individuals afflicted with SMA, it is crucial to evaluate significant factors such as the health-related quality of life (HRQoL). This examination should encompass both the obstacles specific to the disease and the personal burdens it imposes.

At present, the development of innovative treatments for SMA management is progressing rapidly. It's therefore critical to conduct an in-depth study on how these advancements are influencing patients of SMA, particularly in specific cultural settings like Saudi Arabia where high rates consanguineous marriages are prevalent and some cultural beliefs of genetic risk factors.

With this in mind, we aim to provide a more comprehensive understanding of the burden of illness across all types of SMA. The anticipated results could inform and enhance future healthcare planning, benefiting both patients and their caregivers.

## 2 Materials and methods

### 2.1 Study design

This study employed a cross-sectional design, leveraging questionnaires and proxy interviews to survey patients with SMA. The patient pool was selected using a snowball sampling technique from various caregivers attending the neurology clinics at King Khalid University Hospital in Riyadh, Saudi Arabia.

Inclusion in the study was predicated on the patients' confirmed diagnosis of SMA, as reported by their caregivers and verified by their treating neurologists. Exclusion criteria encompassed patients residing outside of Saudi Arabia and cases where caregivers were under the age of 18 years old. In this study, the caregivers of the patients served as proxy respondents, providing crucial data on behalf of the patients they care for.

### 2.2 Data collection

In this study, an interviewer-administered telephone survey was conducted, facilitated by three trained interviewers who engaged in data collection. An interview protocol was devised, and adherence to this protocol was ensured through role-playing exercises, aiming to minimize the risk of interviewer bias. Data collection commenced on July 26, 2022, and concluded on March 21, 2023.

The survey gathered sociodemographic characteristics of patients and caregivers, including factors such as age, gender, educational attainment, monthly income, geographical location, and number of siblings. Additionally, the family history of SMA in patients, the presence of SMA among siblings, the birth order of the patient within their family, and the educational level of the patient were recorded.

Furthermore, patient medical characteristics were documented. This information comprised age at diagnosis, the type of treatment received (supportive care only, Nusinersen, Onasemnogene abeparvovec-xioi, or Risdiplam), dependence on mechanical ventilation, instances of hospitalization in the past year, and participation in a regular physiotherapy program (21).

Out-of-pocket expenditures were assessed using a newly translated Arabic version of the 32-item CoPAQ. This translation adhered to the principles of good practice for the translation and cultural adaptation process for patient-reported outcome measures (22). The CoPAQ includes different questions that inquired about the patient's and caregiver's out-of-pocket expenditures related to the health condition of patient that were not covered by insurance or public assistance, such as, transportation, travels, parking fees, accommodation, prescription and non-prescription medications, dietary supplements, home care services (e.g., rehabilitation), medical devices, home renovation to accommodate patients' health condition, out-of-pocket expenses for healthcare services (e.g., copayment, coinsurance, deductible, and full cost payment for lab test or imaging studies, printing medical reports or certificates, dental services, osteopathy, etc.…), childcare, and pet care.

Patients' quality of life was evaluated using the Arabic version of the EuroQol (EQ-5D-5L) questionnaire among patients aged ≥12 years. Moreover, patients' self-rated health status was assessed using the visual analog scale of EuroQol (EQ-VAS) (23, 24). This version includes five domains: mobility, self-care, usual activities, pain/discomfort, and anxiety/depression. Each domain has five possible levels, providing a comprehensive overview of the HRQoL among SMA patients. Caregivers served as proxy respondents for this assessment.

To gain insight into the mental health of the caregivers, their levels of depression and anxiety were evaluated using the Arabic versions of the Patient Health Questionnaire (PHQ-9) and General Anxiety Disorder-7 (GAD-7) tools, respectively (25, 26). The PHQ-9 consists of 9 items and is widely used in screening individuals on their levels of depression over the last 14 days, while the GAD-7 consists of seven items and screens individuals on their levels of anxiety over the last 14 days (25, 26). High scores in PHQ-9 and GAD-7 scales indicate higher levels of depression and anxiety, respectively.

Lastly, caregivers' health literacy was assessed using the Arabic version of the Single-Item Literacy Screener (SILS). According to this assessment, those who frequently, often, or sometimes required assistance with reading and understanding a medication leaflet were considered to have marginal literacy. Conversely, those who rarely or never needed such help were categorized as having adequate literacy (27, 28).

### 2.3 Data analysis

The requisite minimum sample size was calculated based on the difference in the EQ-VAS score for patients on FDA approved medications for SMA vs. their counterparts on supportive care using an alpha level (α) of 0.05, beta level (β) of 0.2, a large effect size as indicated by Cohen's *d* of 0.8, and a power of 80%. Based on these parameters, the minimum sample size was determined to be 52 SMA patients. Participant characteristics were analyzed using various descriptive statistical measures, including mean, median, standard deviation, interquartile range, as well as frequencies and percentages. Further inferential analysis was conducted using tests such as the Chi-Square test, Fisher's exact test, Student's *t*-test, and Analysis of Variance (ANOVA), as pertinent to the data set. The depression levels of the participants were categorized into five tiers, namely, minimal depression, mild depression, moderate depression, moderately severe depression, and severe depression, based on respective PHQ-9 scores of 5, 10, 15, and 20 (29). Anxiety levels were categorized into four tiers: minimal anxiety (GAD-7 scores 0–4), mild anxiety (GAD-7 scores 5–9), moderate anxiety (GAD-7 scores 10–14), and severe anxiety (GAD-7 scores 15 or above) (30). Only complete data were included in the analysis and no imputation was conducted. Cost in Saudi Riyals (SAR) was converted to United States Dollars (USD) using the fixed currency conversion rate of SAR 3.75 per USD 1. All statistical analyses were executed using the SAS^®^ version 9.4 software suite (SAS Institute, Cary, NC, USA). Graphical representations were generated using Microsoft^®^ Excel 2016.

### 2.4 Ethical considerations

This study received formal approval from the Institutional Review Board (IRB) of King Saud University Medical City (Approval of Research Project No. E-22-6955), located in Riyadh, Saudi Arabia. Strict data access protocols were implemented to ensure the confidentiality and security of the patients' collected data, with access granted solely to the investigators associated with this study. All collected data were stored in a secure and safeguarded location. Personal identifiers, such as national ID numbers, were not collected, further upholding the anonymity of the participants. This research strictly adhered to the ethical principles outlined in the Helsinki Declaration, thereby ensuring the protection and respect of participant rights and welfare.

## 3 Results

### 3.1 Patients' sociodemographic characteristics

A total of 64 caregivers of SMA patients were identified and gave consent to participate in the study. The patients they cared for were divided into SMA types I (*n* = 23), II (*n* = 19), III (*n* = 19), and IV (*n* = 3). The mean patient age was ~13 years. However, patients with type I SMA were significantly younger than those with other types of SMA, with respective mean ages of 3.68 vs. 13.88 years, 22.52 years, and 17.67 years for types II, III, and IV, respectively (*p*-value < 0.0001).

Approximately 53% of the patients were males, and no significant differences were observed in gender distributions across the different types of SMA. All of the participants were Arab and a considerable majority of the patients were of Saudi nationality (93.75%), residing in the three most densely populated regions—Riyadh, Makkah, and the Eastern regions—which accounted for 82.82% of the patients. Furthermore, 85.94% of these patients resided in urban areas.

It was noted that patients with type I SMA had fewer siblings compared to their counterparts with other types of SMA. While the majority of patients did not report a family history of SMA (76.56%), ~48% did have siblings who were also diagnosed with the same type of SMA.

None of the patients with type I SMA were enrolled in formal schools since the majority (78.22%) were under 5 years of age. In contrast, patients with types II, III, and IV were engaged in different levels of formal education (*p*-value < 0.0001). These characteristics are summarized in Table 1.

**Table 1:** Patient baseline characteristics.

**Characteristic**	**Type I**	**Type II**	**Type III**	**Type IV**	***p*-value**	**Total**
	**(*****N*** = **23)**	**(*****N*** = **19)**	**(*****N*** = **19)**	**(*****N*** = **3)**		**(*****N*** = **64)**
**Age**, ***N*** **(%)**						
<1 yr.	5 (21.74)	1 (5.26)	0 (0.0)	0 (0.0)	0.0001^*^	6 (9.38)
≥1– <2 yrs.	4 (17.39)	0 (0.0)	0 (0.0)	0 (0.0)		4 (6.25)
≥2– <5 yrs.	9 (39.13)	2 (10.53)	2 (10.53)	0 (0.0)		13 (20.31)
≥5– <10 yrs.	4 (17.39)	6 (31.58)	4 (21.05)	1 (33.33)		15 (23.44)
≥10– <18 yrs.	0 (0.0)	5 (26.32)	2 (10.53)	0 (0.0)		7 (10.94)
≥18 yrs.	1 (4.35)	5 (26.32)	11 (57.89)	2 (66.67)		19 (29.69)
**Gender**, ***N*** **(%)**						
Male	11 (47.83)	10 (52.63)	11 (57.89)	2 (66.67)	0.9244	34 (53.13)
Female	12 (52.17)	9 (47.37)	8 (42.11)	1 (33.33)		30 (46.88)
**Region**, ***N*** **(%)**						
Riyadh	5 (21.74)	9 (47.37)	4 (21.05)	1 (33.33)	0.4423	19 (29.69)
Makkah	5 (21.74)	5 (26.32)	6 (31.58)	2 (66.67)		18 (28.13)
Eastern region	9 (39.13)	4 (21.05)	3 (15.79)	0 (0.0)		16 (25.00)
Al-Jawf	1 (4.35)	0 (0.0)	0 (0.0)	0 (0.0)		1 (1.56)
Jazan	0 (0.0)	1 (5.26)	2 (10.53)	0 (0.0)		3 (4.69)
Al Qassim	0 (0.0)	0 (0.0)	1 (5.26)	0 (0.0)		1 (1.56)
Aseer	0 (0.0)	0 (0.0)	1 (5.26)	0 (0.0)		1 (1.56)
Tabuk	2 (8.70)	0 (0.0)	2 (10.53)	0 (0.0)		4 (6.25)
Hail	1 (4.35)	0 (0.0)	0 (0.0)	0 (0.0)		1 (1.56)
**Nationality**, ***N*** **(%)**						
Saudi	20 (86.96)	19 (100.0)	18 (94.74)	3 (100.0)	0.6259	60 (93.75)
Non-Saudi	3 (13.05)	0 (0.0)	1 (5.26)	0 (0.0)		4 (6.25)
**Number of siblings**, ***N*** **(%)**						
None	5 (21.74)	0 (0.0)	1 (5.26)	0 (0.0)	0.3251	6 (9.38)
1-2	3 (13.04)	2 (10.53)	2 (10.53)	0 (0.0)		7 (10.94)
3-4	12 (52.17)	9 (47.37)	12 (63.16)	2 (66.67)		35 (54.69)
>4	3 (13.04)	8 (42.11)	4 (21.05)	1 (33.33)		16 (25.00)
**Living in urban or rural areas**, ***N*** **(%)**						
Urban	18 (78.26)	19 (100.00)	15 (78.95)	3 (100.00)	0.1417	55 (85.94)
Rural	5 (21.74)	0 (0.0)	4 (21.05)	0 (0.0)		9 (14.06)
**Family history of SMA**, ***N*** **(%)**						
Yes	4 (17.39)	5 (26.32)	5 (26.32)	1 (33.33)	0.9396	15 (23.44)
No	19 (82.61)	14 (73.68)	14 (73.68)	2 (66.66)		49 (76.56)
**Have siblings with SMA, N (%)**						
Yes	9 (39.13)	12 (63.16)	7 (36.84)	3 (100.0)	0.0906	31 (48.44)
No	14 (60.87)	7 (36.84)	12 (63.16)	0 (0.0)		33 (51.56)
**Which type of SMA your sibling was diagnosed with?**						
SMA type I	6 (26.09)	0 (0.0)	0 (0.0)	0 (0.0)	<0.0001^*^	6 (9.38)
SMA type II	1 (4.35)	9 (47.37)	0 (0.0)	2 (66.66)		12 (18.75)
SMA type III	0 (0.0)	0 (0.0)	6 (31.58)	0 (0.0)		6 (9.38)
SMA type IV	0 (0.0)	0 (0.0)	1 (5.26)	1 (33.33)		2 (3.13)
**Patient order among his/her siblings**, ***N*** **(%)**	2.65 ± 1.55	3.53 ± 2.65	3.05 ± 1.72	4.33 ± 0.58	0.3626	3.11 ± 1.98
First	8 (34.78)	4 (21.05)	5 (26.32)	0 (0.0)	0.1364	17 (26.56)
Second or third	7 (30.43)	8 (42.11)	7 (36.84)	0 (0.0)		22 (34.38)
Fourth or fifth	8 (34.78)	3 (15.79)	6 (31.58)	3 (100.0)		20 (31.25)
Sixth or more	0 (0.0)	4 (21.05)	1 (5.26)	0 (0.0)		5 (7.81)
**Patient current educational level**						
Not in school	23 (100.00)	4 (21.05)	3 (11.11)	0 (0.00)	<0.0001^*^	28 (43.75)
Kindergarten	0 (0.00)	0 (0.00)	1 (5.26)	0 (0.00)		2 (3.13)
Elementary	0 (0.00)	10 (52.63)	4 (21.05)	1 (33.33)		15 (23.44)
Intermediate	0 (0.00)	1 (5.26)	1 (5.26)	0 (0.00)		3 (4.69)
Secondary	0 (0.00)	2 (10.53)	4 (21.05)	1 (33.33)		7 (10.94)
College	0 (0.0)	2 (10.53)	6 (31.58)	1 (33.33)		9 (14.06)

^*^p-value <0.05.

### 3.2 Patients' medical characteristics

As expected, patients with SMA type I were diagnosed at a younger age compared to those with other types of SMA (1.29 vs. 3.29 years, 8.78 years, and 10.33 years for types II, III, and IV, respectively, *p*-value = 0.0003).

About 52% of patients were administered FDA-approved orphan medicinal products specific to SMA treatment, such as Nusinersen, Onasemnogene abeparvovec-xioi, and Risdiplam. Among these, the vast majority (90.91%) were treated with Nusinersen.

A significant proportion of type I patients (87%) were dependent on mechanical ventilation, contrasting with only 15.79% of patients with type II SMA, and none with types III and IV (*p*-value <0.0001). Similarly, the majority of type I patients had been hospitalized during the previous 12 months, compared to 47.37, 26.32, and 0.0% for types II, III, and IV, respectively (*p*-value <0.0001).

**Figure 1:**
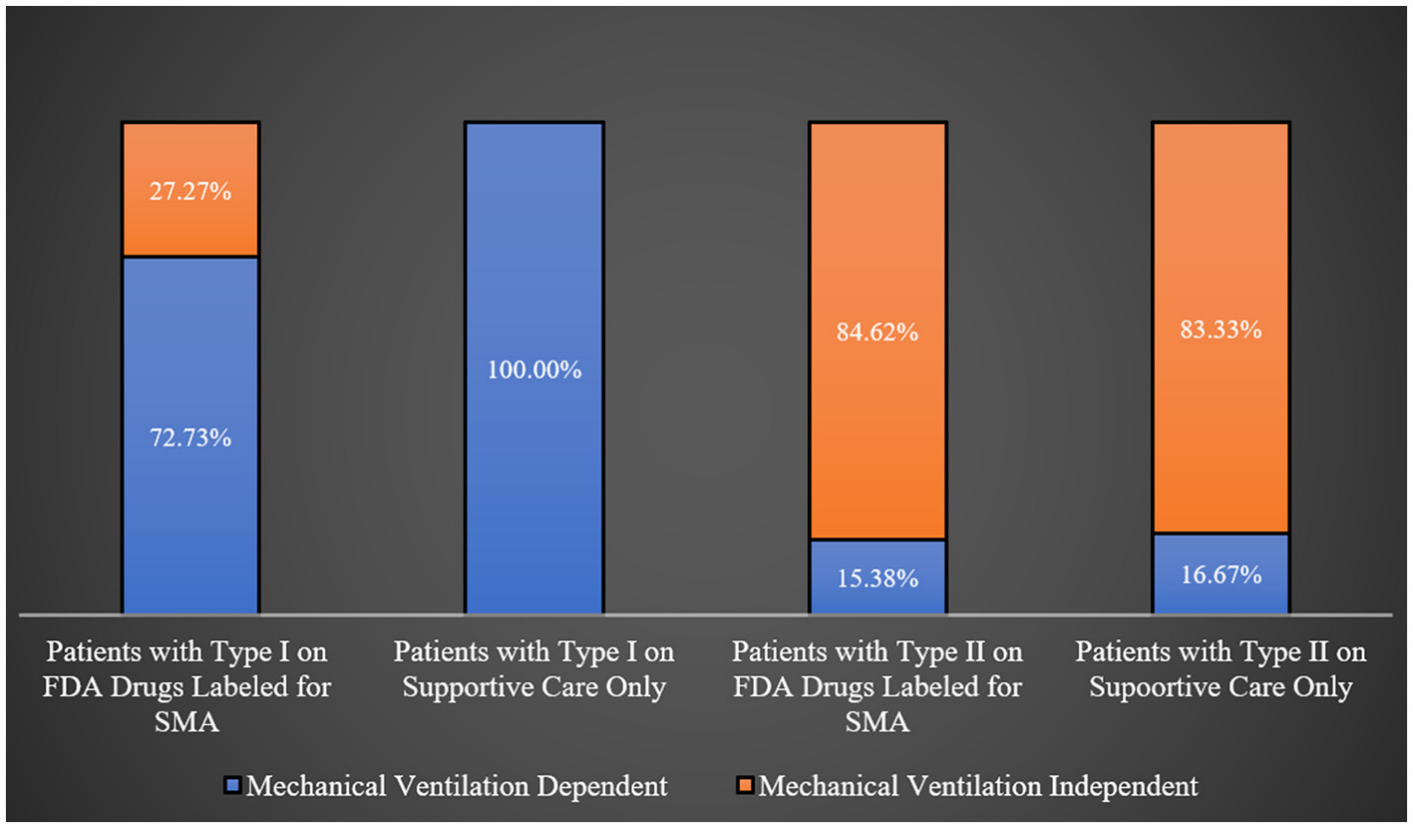
Mechanical ventilation status across patients on different treatment regimens.

Most patients with types I, II, and IV were engaged in regular physical therapy programs, whereas a majority of type III patients were not (68.42%; *p*-value = 0.0181) as shown in Table 2. Interestingly, all type I SMA patients on supportive care were ventilator dependent, in comparison to 72.73% of those treated with FDA-approved orphan medical products for SMA. However, this difference was not statistically significant (*p*-value = 0.0932). Similarly, there was no noticeable difference in the percentages of ventilator-dependent patients among type II SMA patients based on the type of treatment (supportive care only vs. FDA-approved drugs for SMA; *p*-value = 1.0000). This is graphically represented in Figure 1.

**Table 2:** Patient medical characteristics.

**Characteristic**	**Type I**	**Type II**	**Type III**	**Type IV**	***p*-value**	**Total**
	**(*****N*** = **23)**	**(*****N*** = **19)**	**(*****N*** = **19)**	**(*****N*** = **3)**		**(*****N*** = **64)**
**Age at diagnosis (yrs.), mean** **±SD**	1.29 ± 2.16	3.29 ± 3.89	8.78 ± 7.94	10.33 ± 9.01	0.0003^*^	4.54 ± 6.16
**Type of treatment received**, ***N*** **(%)**						
Only supportive care	12 (52.17)	6 (31.58)	10 (52.63)	3 (100.0)	0.3243	31 (48.44)
Nusinersen	9 (39.13)	12 (63.16)	9 (47.37)	0 (0.0)		30 (46.88)
Onasemnogene abeparvovec-xioi	1 (4.35)	1 (5.26)	0 (0.0)	0 (0.0)		2 (3.13)
Risdiplam	1 (4.35)	0 (0.0)	0 (0.0)	0 (0.0)		1 (1.56)
**Patient is dependent on mechanical ventilation**, ***N*** **(%)**						
No	3 (13.04)	16 (84.21)	19 (100.0)	3 (100.0)	<0.0001^*^	41 (64.06)
Yes	20 (86.96)	3 (15.79)	0 (0.0)	0 (0.0)		23 (35.94)
**Rates of hospitalization in the past 12 months**, ***N*** **(%)**						
Yes	21 (91.30)	9 (47.37)	5 (26.32)	0 (0.0)	<0.0001^*^	35 (54.69)
No	2 (8.70)	10 (52.63)	14 (73.68)	3 (100.0)		29 (45.31)
**Rates of intensive care unit (ICU) hospitalization in the past 12 months**, ***N*** **(%)**						
Yes	19 (82.61)	9 (47.37)	5 (26.32)	0 (0.0)	0.0004^*^	33 (51.56)
No	4 (17.39)	10 (52.63	14 (73.68)	3 (100.0)		31 (48.44)
**Patient is enrolled in regular physical therapy?** ***N*** **(%)**						
Yes	15 (65.22)	15 (78.95)	6 (31.58)	2 (66.67)	0.0181^*^	38 (59.38)
No	8 (34.78)	4 (21.05)	13 (68.42)	1 (33.33)		26 (40.63)

^*^p-value <0.05.

There were no significant differences in the rates of hospitalization in the past 12 months among SMA type I patients on supportive care only or those on FDA-approved drugs for SMA (91.67 vs. 90.91%, *p*-value = 0.9486). Even though the rates of hospitalization in the past 12 months among patients with types II and III SMA treated with FDA-approved drugs for SMA were higher than their counterparts on supportive care only (53.85 vs. 33.33% and 33.33 vs. 20.00%, respectively), this difference did not reach statistical significance (*p*-value = 0.6285).

### 3.3 Proxy-reported health-related quality of life

The number of patients aged ≥12 years who answered the EQ-5D-5L questions on the five domains were 22 patients. All patients with SMA types I and II, according to their EQ-5D-5L responses, were incapable of walking, contrasting sharply with only 50.0% of type III patients and none of the type IV patients. Similarly, all of the patients with type I and II were unable to take care of themselves and the majority were unable to perform their usual activities as shown in Table 3.

**Table 3:** Proxy-reported EuroQol-5-D-5-L scores for those aged 12 years and above.

**Characteristic**	**Type I**	**Type II**	**Type III**	**Type IV**	***p*-value**	**Total**
	**(*****N*** = **1)**	**(*****N*** = **9)**	**(*****N*** = **10)**	**(*****N*** = **2)**		**(*****N*** = **22)**
**EQ-5D-5L domains**						
**Mobility**, ***N*** **(%)**						
I have no problems in walking about	0 (0.0)	0 (0.0)	1 (10.0)	2 (100.0)	0.6015	3 (13.64)
I have slight problems in walking about	0 (0.0)	0 (0.0)	2 (20.0)	0 (0.0)		2 (9.09)
I have moderate problems in walking about	0 (0.0)	0 (0.0)	1 (10.0)	0 (0.0)		1 (4.55)
I have severe problems in walking about	0 (0.0)	0 (0.0)	1 (10.0)	0 (0.0)		1 (4.55)
I am unable to walk about	1 (100.0)	9 (100.0)	5 (50)	0 (0.0)		15 (68.18)
**Self-care**, ***N*** **(%)**						
I have no problems washing or dressing myself	0 (0.0)	0 (0.0)	2 (20.00)	0 (0.0)	0.4299	2 (9.09)
I have slight problems washing or dressing myself	0 (0.0)	0 (0.0)	2 (20.00)	0 (0.0)		2 (9.09)
I have moderate problems washing or dressing myself	0 (0.0)	0 (0.0)	2 (20.00)	2 (100.0)		4 (18.18)
I have severe problems washing or dressing myself	0 (0.0)	0 (0.0)	1 (10.00)	0 (0.0)		2 (9.09)
I am unable to wash or dress myself	1 (100.0)	9 (100.0)	3 (30.00)	0 (0.0)		13 (59.09)
**Usual activities (e.g., work, study, housework, family, or leisure activities)**, ***N*** **(%)**						
I have no problems doing my usual activities	0 (0.0)	0 (0.0)	1 (10.00)	0 (0.0)	0.0425	1 (4.55)
I have slight problems doing my usual activities	0 (0.0)	0 (0.0)	3 (30.00)	2 (100.0)		5 (22.73)
I have moderate problems doing my usual activities	0 (0.0)	0 (0.0)	2 (20.00)	0 (0.0)		2 (9.09)
I have severe problems doing my usual activities	0 (0.0)	2 (22.22)	2 (20.00)	0 (0.0)		4 (18.18)
I am unable to do my usual activities	1 (100.0)	7 (77.78)	2 (20.00)	0 (0.0)		10 (45.45)
**Pain/discomfort**, ***N*** **(%)**						
I have no pain or discomfort	0 (0.0)	1 (11.11)	4 (40.00)	0 (0.0)	0.1718	5 (22.73)
I have slight pain or discomfort	1 (100.0)	4 (44.44)	1 (10.00)	2 (100.0)		8 (36.36)
I have moderate pain or discomfort	0 (0.0)	4 (44.44)	2 (20.00)	0 (0.0)		6 (26.92)
I have severe pain or discomfort	0 (0.0)	0 (0.0)	1 (10.00)	0 (0.0)		1 (4.55)
I have extreme pain or discomfort	0 (0.0)	0 (0.0)	2 (20.00)	0 (0.0)		2 (9.09)
**Anxiety/depression**, ***N*** **(%)**						
I am not anxious or depressed	1 (100.00)	5 (55.56)	6 (60.00)	0 (0.0)	0.5237	12 (54.55)
I am slightly anxious or depressed	0 (0.0)	2 (22.22)	1 (10.00)	2 (100.0)		5 (22.73)
I am moderate anxious or depressed	0 (0.0)	2 (22.22)	1 (10.00)	0 (0.0)		3 (13.64)
I am severely anxious or depressed	0 (0.0)	0 (0.0)	1 (10.00)	0 (0.0)		1 (4.55)
I am extremely anxious or depressed	0 (0.0)	0 (0.0)	1 (10.00)	0 (0.0)		1 (4.55)

**Figure 2:**
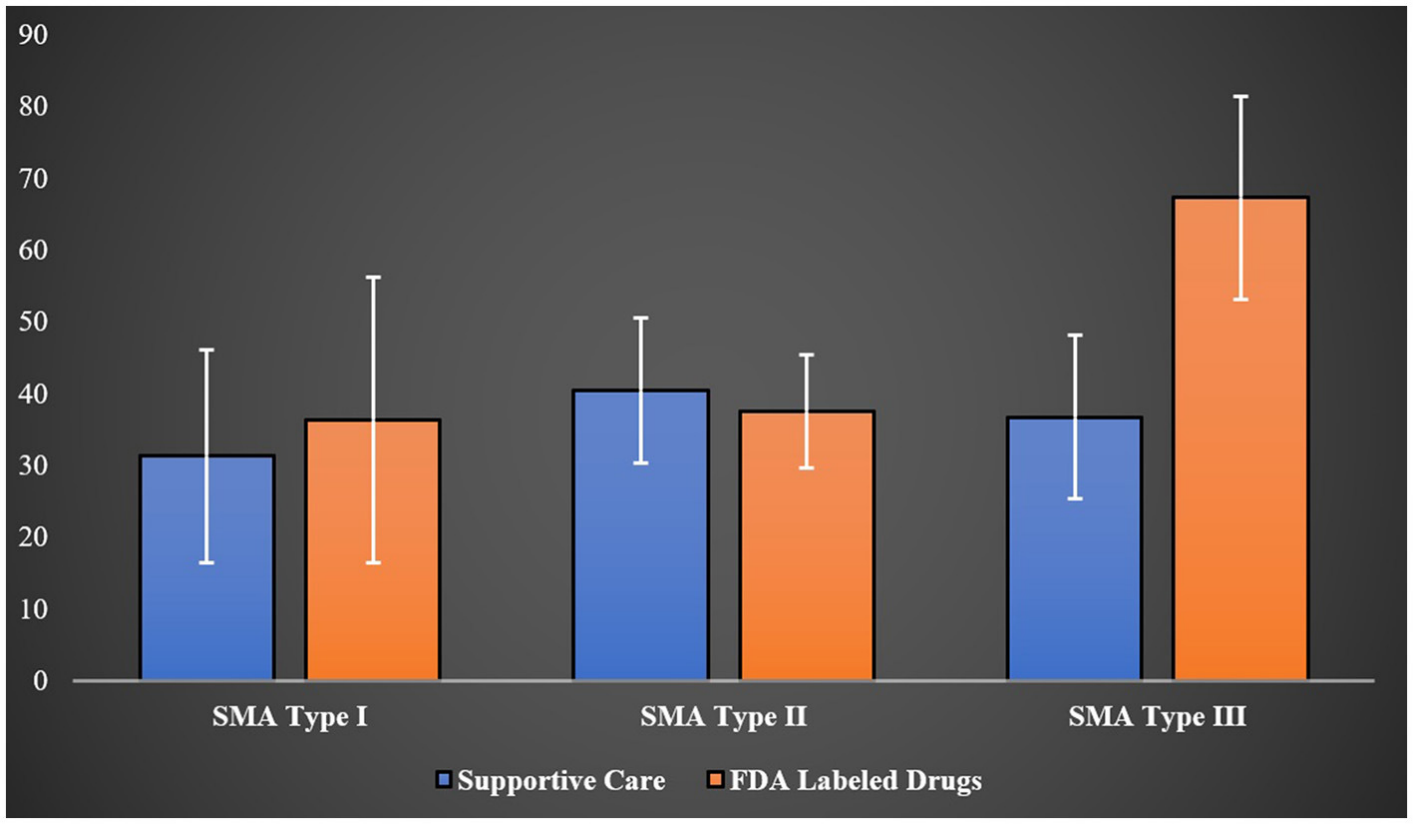
The EQ-VAS scores for patients with type I, II, and III of SMA.

Utilizing the EQ-VAS, which assessed overall health on a scale from 0 (worst imaginable health) to 100 (best imaginable health) for 52 SMA patients from different age groups (type I = 16, type II = 17, type III = 16, type IV = 3), the mean scores were 33.85 for type I, 38.57 for type II, 52.04 for type III, and 40.66 for type IV patients (*p*-value = 0.0137). Interestingly, when EQ-VAS scores were stratified across SMA types and treatment (supportive care only vs. treatment with FDA-approved drugs for SMA), the higher mean score observed in type III patients compared to other types of SMA was no longer apparent. However, type III patients managed solely with supportive care such as nutritional and respiratory support registered a mean EQ-VAS score of 36.75 ± 11.40, compared to 67.33 ± 14.15 among those treated with FDA-approved drugs for SMA (*p*-value <0.0001) as demonstrated in Figure 2.

### 3.4 Caregivers' baseline characteristics and financial burden

In this study, the majority of caregivers for SMA patients were found to be parents (82.18%), married (90.63%), between the ages of 20 and 30 (75.01%), holding an associate degree or higher (51.56%), and possessing adequate health literacy (65.63%). Interestingly, 53.13% of the caregivers did not have a paid job, and most were earning <$1,600 a month (i.e., this includes the main caregiver's income and does not include household income) which is deemed below the average national monthly income (e.g., USD 2,730). A significant majority (85.94%) did not receive any formal education or training on how to provide care for SMA patients, and 59.38% traveled to seek medical consultation for their patient(s) with SMA. Even so, 93.75% did not experience income loss (for example, from missing work days due to their patient's illness), as highlighted in Table 4. Nevertheless, more than half of the caregivers reported experiencing some form of financial stress.

**Table 4:** Caregivers' characteristics and SMA financial burden.

**Characteristic**	**Type I**	**Type II**	**Type III**	**Type IV**	***p*-value**	**Total**
	**(*****N*** = **23)**	**(*****N*** = **19)**	**(*****N*** = **19)**	**(*****N*** = **3)**		**(*****N*** = **64)**
**Relationship of main caregiver to patient**, ***N*** **(%)**						
Mother	13 (56.52)	10 (52.63)	12 (63.16)	1 (33.33)	0.0522	36 (56.25)
Father	10 (43.48)	4 (21.05)	2 (10.53)	1 (33.33)		17 (26.56)
Sibling	0 (0.0)	2 (10.53)	2 (10.53)	0 (0.0)		4 (6.25)
Spouse	0 (0.0)	1 (5.26)	2 (10.53)	1 (33.33)		4 (6.25)
Maid^†^	0 (0.0)	2 (10.53)	1 (5.26)	0 (0.0)		3 (4.69)
**Marital status of the main caregiver**, ***N*** **(%)**						
Single	0 (0.00)	1 (5.26)	1 (5.26)	0 (0.00)	0.0524	2 (3.13)
Married	23 (100.0)	15 (78.95)	17 (89.47)	3 (100.0)		58 (90.63)
Divorced	0 (0.00)	3 (15.79)	1 (5.26)	0 (0.00)		4 (6.25)
**Age of the main caregiver**, ***N*** **(%)**						
<20 yrs.	2 (8.69)	0 (0.0)	0 (0.0)	0 (0.0)	0.1618	2 (3.13)
20–30 yrs.	6 (26.09)	2 (10.53)	2 (10.53)	0 (0.0)		10 (15.63)
31–40 yrs.	14 (60.87)	12 (63.16)	9 (47.37)	3 (100.0)		38 (59.38)
41–50 yrs.	1 (4.35)	3 (15.79)	4 (21.05)	0 (0.0)		8 (12.5)
51–60 yrs.	0 (0.0)	2 (10.53)	3 (15.79)	0 (0.0)		5 (7.81)
61–70 yrs.	0 (0.0)	0 (0.0)	1 (5.26)	0 (0.0)		1 (1.56)
**Educational level of the caregiver**, ***N*** **(%)**						
No formal education	0 (0.0)	0 (0.0)	1 (5.26)	0 (0.0)	0.4367	1 (1.56)
Elementary school	3 (13.04)	0 (0.0)	2 (10.53)	0 (0.0)		5 (7.81)
Intermediate school	0 (0.0)	1 (5.26)	0 (0.0)	0 (0.0)		1 (1.56)
High school diploma	10 (43.48)	5 (26.32)	9 (47.37)	0 (0.0)		24 (37.5)
Associate degree	2 (8.69)	1 (5.26)	1 (5.26)	0 (0.0)		4 (6.25)
College degree	6 (26.09)	11 (57.89)	6 (31.58)	3 (100.0)		26 (40.63)
Postgraduate degree	2 (8.69)	1 (5.26)	0 (0.0)	0 (0.0)		3 (4.69)
**Health literacy**, ***N*** **(%)**						
Adequate	12 (52.17)	16 (84.21)	12 (63.16)	2 (66.67)	0.1642	42 (65.63)
Marginal	11 (47.83)	3 (15.79)	7 (36.84)	1 (33.33)		22 (34.38)
**Do you have a paying job?**						
Yes	11 (47.83)	11 (57.89)	7 (36.84)	1 (33.33)	0.4973	30 (46.88)
No	12 (52.17)	8 (42.11)	12 (63.16)	2 (66.66)		34 (53.13)
**Monthly income (USD)**, ***N*** **(%)**						
$0–$800	12 (52.17)	8 (42.11)	9 (47.37)	3 (100.0)	0.5497	32 (50.0)
$800–$1,600	5 (21.74)	3 (15.79)	3 (15.79)	0 (0.0)		11 (17.19)
$1,600–$2,666.67	3 (13.04)	1 (5.26)	3 (15.79)	0 (0.0)		7 (10.94)
$2,666.67–$4,000	1 (4.35)	3 (15.79)	2 (10.53)	0 (0.0)		6 (9.38)
$4,000–$5,333.33	1 (4.35)	3 (15.79)	1 (5.26)	0 (0.0)		5 (7.81)
>$5,333.33	1 (4.35)	1 (5.26)	0 (0.0)	0 (0.0)		2 (3.125)
**Did you receive education or training sessions on how to care for SMA patients?** ***N*** **(%)**						
Yes	6 (26.09)	1 (5.26)	2 (10.53)	0 (0.0)	0.2434	9 (14.1)
No	17 (73.91)	18 (94.74)	17 (89.47)	3 (100.0)		55 (85.94)
**Did you travel to seek medical consultation for your relative?** ***N*** **(%)**						
Yes	13 (56.52)	10 (52.63)	14 (73.68)	1 (33.33)	0.2038	38 (59.38)
No	10 (43.48)	9 (47.37)	5 (26.32)	2 (66.66)		26 (40.63)
**Did you suffer any income loss due to your relative's disease?** ***N*** **(%)**						
Yes	2 (8.70)	2 (10.53)	0 (0.0)	0 (0.0)	0.7389	4 (6.25)
No	21 (91.30)	17 (89.47)	19 (100.0)	3 (100.0)		60 (93.75)
**What was the reason behind the income loss?** ***N*** **(%)**						
Missed days or showing up late to work	2 (8.70)	1 (5.26)	0 (0.0)	0 (0.0)	1.00	3 (4.69)
Health insurance refusal to pay	0 (0.0)	1 (5.26)	0 (0.0)	0 (0.0)		1 (1.56)
**Are you under financial strain?** ***N*** **(%)**						
Refrain from answering	5 (21.74)	4 (21.05)	8 (42.11)	1 (33.33)	0.0345	18 (28.13)
Not at all	4 (17.39)	2 (10.53)	6 (31.58)	0 (0.0)		12 (18.75)
Sometimes	6 (26.09)	3 (15.79)	0 (0.0)	2 (66.66)		11 (17.19)
Often	3 (13.04)	5 (26.32)	0 (0.0)	0 (0.0)		8 (12.5)
Always	5 (21.74)	5 (26.32)	5 (26.32)	0 (0.0)		15 (23.44)
**Patient Health Questionnaire-9 (PHQ-9), Mean** **±SD**	9.88 ± 4.77	6.65 ± 4.95	6.38 ± 3.92	3.00 ± 3.58	0.0155^*^	7.56 ± 4.79
**Generalized Anxiety Disorder-7 (GAD-7), Mean** **±SD**	6.79 ± 5.45	4.18 ± 4.30	5.38 ± 4.72	6.0 0 ± 4.97	0.3803	5.56 ± 4.81
**Estimated total out of pocket expenditures since the time of diagnosis, mean** **±SD (USD)**	3,722.32 ± 6,087.38	13,492.49 ± 15,799.45	33,366.32 ± 73,775.52	3,111.11 ± 2,694.30	0.1399	15,394.75 ± 42,367.53

^†^Maids who are part of the household served as proxy respondents to the biological main caregivers (e.g., father, mother, sister, and brother) due to the difficulty of reaching the biological relatives most of the time. ^*^p-value <0.05.

**Figure 3:**
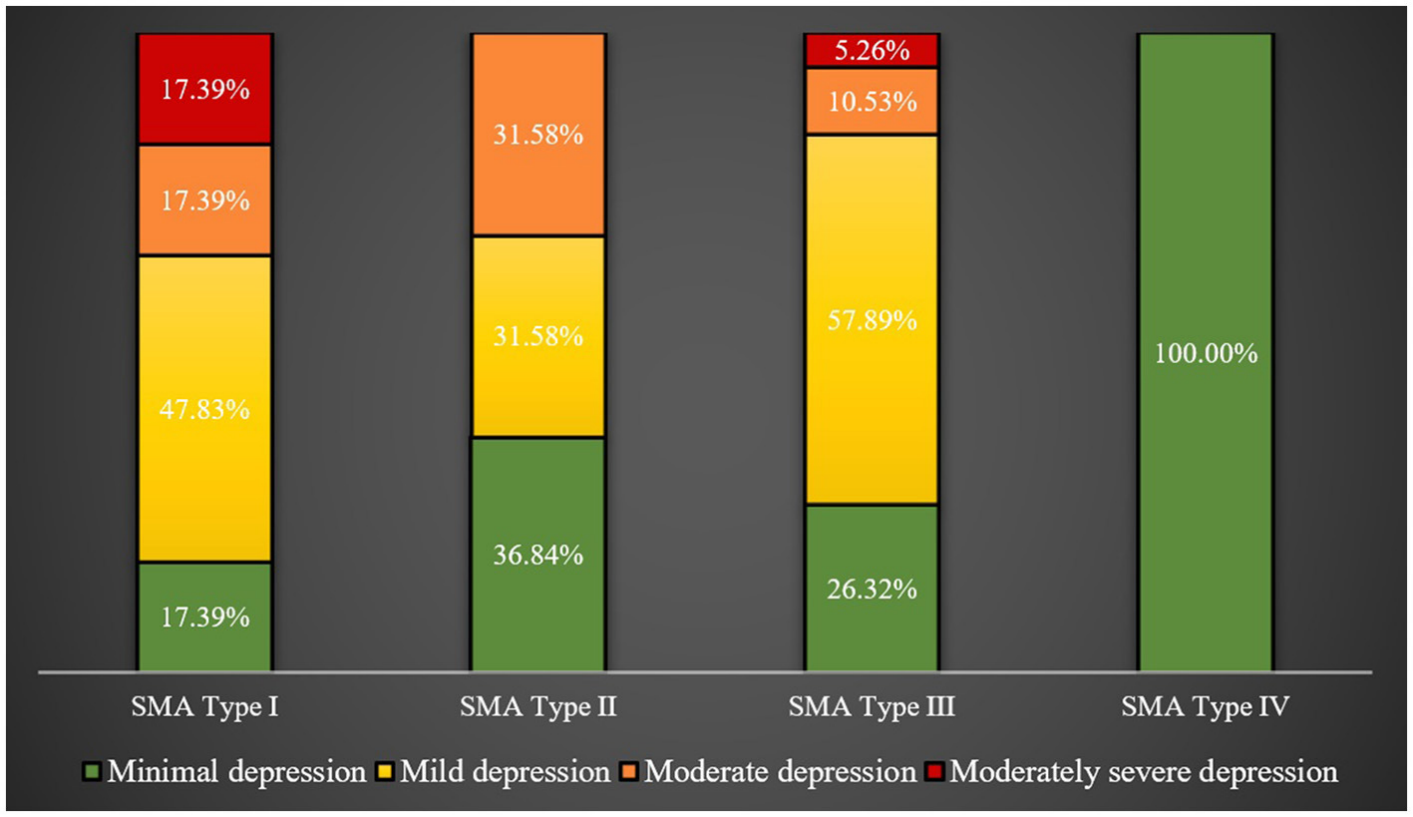
Rates of depression across caregivers of patients with different SMA types.

The caregivers for SMA type I patients, followed by those for type II patients, had significantly higher mean PHQ-9 scores than caregivers for other types of SMA patients (*p*-value = 0.0155). This is visibly demonstrated in Figure 3, showing 34.78 and 31.58% of caregivers for SMA type I and II patients, respectively, experiencing moderate to moderately severe depression, compared to just 15.79% of caregivers for type III patients.

**Figure 4:**
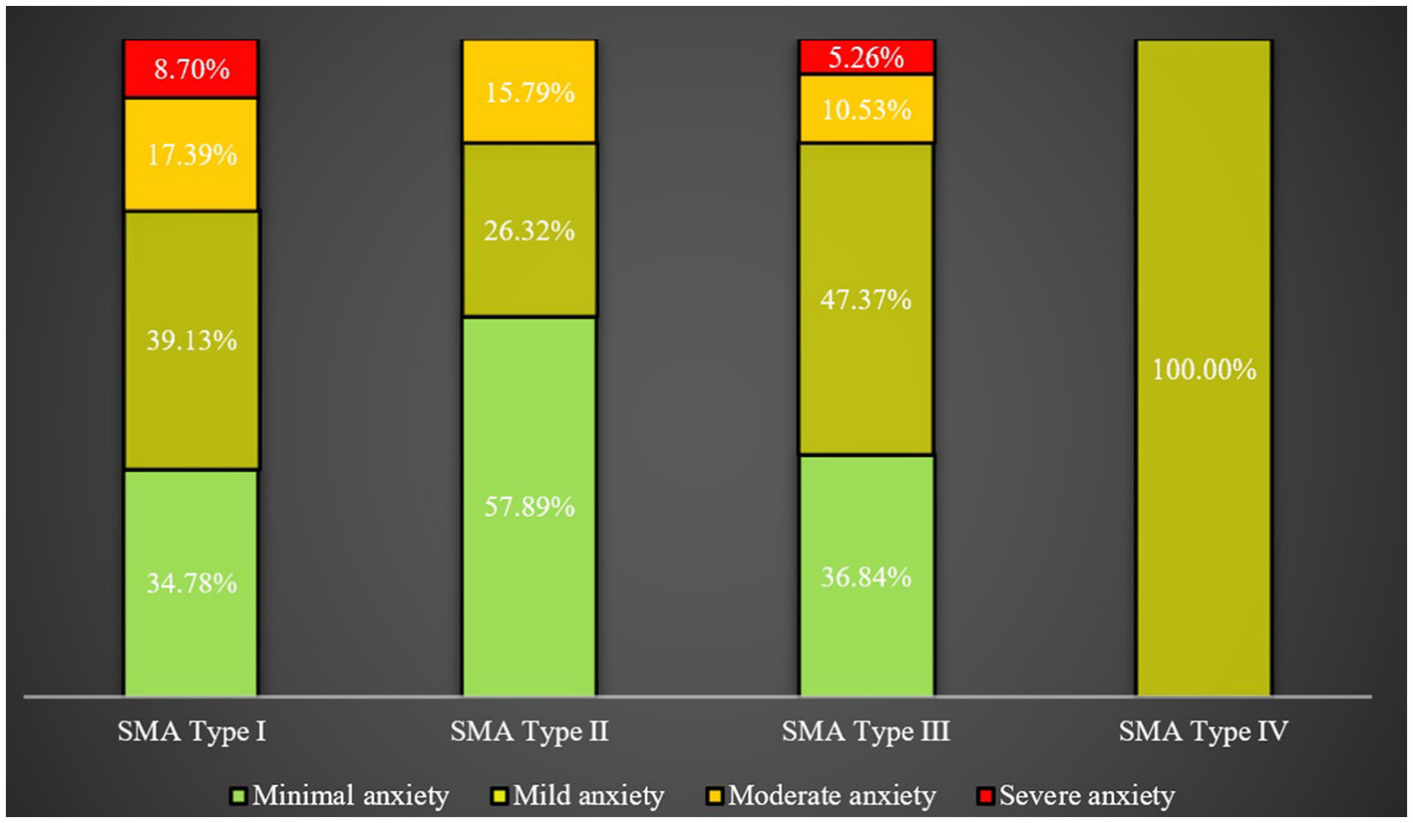
Rates of anxiety across caregivers of patients with different SMA types.

However, no significant difference was detected in the mean GAD-7 scores among caregivers for patients with different types of SMA, nor in levels of anxiety, as illustrated in Figure 4.

Interestingly, caregivers for SMA type III patients reported the highest mean out-of-pocket expenditures. The mean total out-of-pocket expenditures per patient per year amounted to USD 7,099.77 for type I, USD 3,395.75 for type II, USD 19,055.58 for type III, and USD 444.45 for type IV. These expenses encompassed both medical costs (for instance, private clinic visits, over-the-counter medications, and medical devices) and non-medical costs (such as home renovations or purchasing a specially-equipped vehicle).

## 4 Discussion

To the best of our knowledge, this study represents the first attempt to investigate the socioeconomic burden experienced by both SMA patients and their caregivers in Saudi Arabia. By delving into the various aspects of the socioeconomic impact of SMA, we aimed to contribute valuable insights to the existing body of knowledge in this field.

Although a previously published study examined the impact of Nusinersen treatment on the HRQoL of SMA patients in Saudi Arabia, using caregivers as proxy respondents, raised concerns regarding the effectiveness of Nusinersen in improving HRQoL, this study did not assess the psychological impact of SMA on caregivers or considered the financial burden associated with the illness. Consequently, our research comprehensively evaluated the psychological wellbeing of caregivers and quantify the out-of-pocket expenditures related to SMA management (31).

Moreover, the economic burden of SMA and the cost-effectiveness of treatments have been evaluated in different countries. These studies provide insights into the direct medical and non-medical costs associated with SMA, as well as the impact on patients and caregivers.

A systematic review conducted in 2020 identified a range of cost-effectiveness evaluations for SMA treatments. The incremental cost-effectiveness ratio (ICER) for nusinersen, one of the treatments assessed, varied from $210,095 to $1,150,455 per quality-adjusted life years (QALY) gained (14). Furthermore, another study found that the mean per-patient annual direct medical costs ranged from $3,320 to $324,410, depending on the type of SMA and the country (19).

In terms of the burden on patients and caregivers, a study conducted in Canada highlighted the impaired quality of life experienced by SMA patients. Caregivers reported the need for various forms of support, such as assistive devices and health professional services. They also faced challenges in terms of personal plans, sleep disturbances, and work adjustments (20). Similarly, a study in Hong Kong revealed a high healthcare burden and cumulative life costs for SMA patients, particularly those with type 1 and type 2 SMA, who did not receive disease-modifying treatment (32).

In our own research conducted in Saudi Arabia, we observed specific patterns within different SMA types. Type 1 patients had higher rates of sibling concordance, ICU hospitalization, and a greater need for mechanical support. Conversely, Type 3 patients exhibited a better quality of life. Type 1 patients' caregivers also showed higher depression scores, while caregivers of Type 3 patients reported higher out-of-pocket expenditures. The higher out-of-pocket expenditures reported by caregivers for patients with type III SMA is expected due to the higher survival rates compared to patients with types I and II and the earlier onset of the disease compared to patients with type IV (6–8). Moreover, it is worth noting that 48% of patients received supportive care, while others received SMA therapies. The overall findings underscored the significant socioeconomic burden imposed by SMA on patients and caregivers, emphasizing the need for increased attention from the healthcare system. Access to innovative therapies varied across different SMA types. Therefore, further efforts should be directed toward implementing screening programs and providing timely access to innovative therapies to mitigate the impact of SMA on individuals and society. These findings underscore the importance of providing patients with SMA with supplemental health insurance to cover other expenses that are mostly uncovered by the public healthcare coverage such as durable medical equipment. Additionally, there are other non-medical expenses, such as, vehicle modifications to accommodate needs of patients with disabilities and home remodeling for people's disabilities. Therefore, patients with rare and burdensome illnesses, such as SMA, should receive supplemental government financial support.

On the other hand, significant variation in the mean age across different subtypes of SMA was observed. Notably, the mean age for type III was observed to be higher in comparison to type I (22.52 ± 15.97 vs. 3.68 ± 5.16). These results align with previous studies that have consistently reported a mean age of onset of 5 months for SMA type 1 patients, 11.5 months for type 2, 4.5 years for type 3, and 18 years for type 4 (33, 34).

Furthermore, it is worth noting that the mean age for type IV was found to be 17.67 ± 8.50. This discrepancy may be attributed to the relatively small number of patients with type IV SMA in our study sample, as there were only three participants compared to the other SMA types that had nearly 20 participants. Alternatively, this variation could potentially reflect the natural diversity present within the Saudi population. However, it is important to emphasize that a larger sample size in prospective studies would be needed to obtain more conclusive insights into this matter.

The age distribution corresponded with the age of diagnosis and educational level. Among SMA type I patients, the majority were children who had not yet started school. Similarly, in type II, most individuals were either in elementary school or had completed it. Interestingly, type III exhibited a wide distribution, with some patients as early as kindergarten and others already in college. However, this distribution does not necessarily align with age and may simply reflect the challenges faced by individuals with SMA in pursuing education.

Furthermore, this study revealed a notable trend in the Saudi population, where siblings tended to share the same subtype of SMA, as expected in a disease with a familial distribution. Similar findings have been reported in other studies. For instance, a cohort study examining 303 siblings identified between 1996 and 2016 reported that 84.8% of them exhibited subtype concordance. The distribution of concordant subtypes in this study was as follows: Type I, 54.5%; Type II, 31.9%; Type III, 13.2%; Type IV, 0.4% (35). These findings provide further evidence of the familial nature of SMA and its impact on affected siblings.

SMA type 1 patients constituted the majority of individuals requiring mechanical ventilation (86.96% vs. 3% vs. 0% vs. 0%) due to the severe nature of the disease and its early onset at around 6 months of age. These patients typically experience a rapid disease course, leading to lifelong reliance on ventilatory support before the age of 2 (36). Consequently, it is not surprising that a significant proportion of SMA type 1 patients had a history of hospitalization (91.3% vs. 47.37% vs. 26.32% vs. 0%). Among the 21 SMA type 1 patients requiring mechanical ventilation, 19 of them were admitted to the intensive care unit (ICU). It is worth noting that current treatment guidelines for SMA, particularly types 1–3, emphasize the importance of early assessment of lung function and the implementation of supportive respiratory therapies (37). These include techniques such as air stacking, physiotherapy, mechanical insufflation, and mechanical exsufflation procedures. While patients with other subtypes may not have utilized mechanical ventilatory support, they may have employed less invasive respiratory devices like CPAP or oxygen tanks. However, this specific information was not collected from the study participants.

The majority of individuals across all SMA subtypes engaged in physiotherapy, regardless of disease severity, which is crucial for maximizing physical functionality. Physiotherapy helps improve posture, prevent joint immobility, and reduce muscle atrophy and weakness (38, 39). Guidelines recommend that all SMA patients have access to specialized neuromuscular centers, where they can receive regular evaluations and physiotherapy recommendations from skilled and experienced professionals every 6 months (40, 41). However, it is concerning that ~41% of the study sample were not enrolled in regular physical therapy, as this may lead to worse clinical outcomes and a faster rate of disease progression, even among those receiving medications with FDA-labeled indications for SMA (42).

The availability of specialized physical therapy centers catering to SMA patients in Saudi Arabia is insufficient, and it is imperative for the Saudi Physical Therapy Association (SPTA) to prioritize this specific patient subset while promoting awareness about the prevalence of the disease within the country.

According to the proxy-reported EuroQol assessment, the HRQoL demonstrated improvement with increasing SMA subtype, with Type I and Type II patients reporting the lowest HRQoL, while Type III and Type IV patients showed relatively better HRQoL based on the EQ-5D-5-L and EQ-VAS scales.

In terms of mobility and self-care, all participants with Type I and Type II SMA were unable to walk or maintain self-care. Additionally, all Type I individuals reported an inability to perform usual daily activities, compared to 76.47% of Type II patients. On the other hand, ~50% of Type III patients had either no problem, slight problem, or moderate problem in walking. These findings are consistent across the four other domains of the EQ-5D-5L questionnaire, except for anxiety/depression. Similar studies have shown better HRQoL among patients with Type III SMA compared to their counterparts with Type I and Type II SMA (43). Pain symptoms were not widespread, with only 43.75% of Type I patients reporting severe pain. While there was no significant association between anxiety/depression and SMA subtype or symptom severity, overall EQ-VAS values were higher for Type III and Type IV patients. Interestingly, another study conducted in Iran found no significant difference in HRQoL between Type II and Type III SMA patients (44).

The psychological wellbeing of caregivers showed that the majority experienced minimal to mild levels of anxiety and depression, regardless of the type of patient they were caring for. However, caregivers for patients with type I SMA exhibited higher PHQ-9 scores, which is intriguing considering that previous studies examining the psychological impact on caregivers of SMA patients often reported elevated levels of depression and anxiety (45, 46). This observation may be attributed to the cultural concept of filial piety, as Saudi Arabian culture and religion place significant emphasis on caring for the sick and older adults, regarding it as a noble and rewarding act (47).

Analysis of out-of-pocket expenditures incurred by SMA caregivers since the time of diagnosis revealed higher costs for type III, followed by type II, compared to type I. This is primarily due to the higher survival rates associated with type III and II SMA, in contrast, to type I, which typically has a life expectancy of <2 years (48). Additionally, it is worth noting that a significant portion of the out-of-pocket expenditures were not directly medically related. These expenses included purchasing care for individuals with special needs and making home renovations to accommodate the needs of the patients. This pattern may be influenced by the presence of universal healthcare coverage for citizens in Saudi Arabia, resulting in fewer medically-related out-of-pocket costs (49).

Regarding treatment options, all three therapies approved by the United States Food and Drug Administration (Nusinersen, Onasemnogene abeparvovec-xioi, and Risdiplam) have also been approved by the Saudi Food and Drug Authority (SFDA). However, the access to these therapies varied among the study participants, with Nusinersen being the most commonly utilized SMA therapy, accounting for ~47% of cases. This discrepancy in utilization rates may be attributed to the fact that Nusinersen was the first SMA therapy approved by the USFDA in December 2016, while Onasemnogene abeparvovec-xioi received approval in May 2019, and Risdiplam in August 2020 (50).

Moreover, these findings shed light on the psychological wellbeing of caregivers, the financial burden they face, and the availability of SMA therapies in Saudi Arabia. Understanding these aspects is crucial for developing support systems and interventions that cater to the specific needs of caregivers and patients with SMA in the country.

Interestingly, no significant difference was observed in the rates of patients on mechanical ventilation or the rate of hospitalization in the past 12 months between patients managed with FDA-labeled therapies and those managed with supportive care alone. This finding aligns with a previously published study conducted in Saudi Arabia that reported similar results (31).

However, among SMA patients with type III, those managed with Nusinersen showed a significantly higher mean EQ-VAS score, which measures overall health-related quality of life (HRQoL), compared to those managed with supportive care alone. Conversely, no significant difference was found between patients with type I and II managed with FDA-labeled therapies or supportive care. These findings are intriguing, as studies assessing HRQoL among patients managed with Nusinersen have not consistently demonstrated significant improvements across different SMA types, despite modest improvements observed in type I and II cases (31, 51, 52). However, these findings align with a recently published multicenter study conducted in Italy, which evaluated the impact of Nusinersen treatment on a group of adult SMA patients, including 69 individuals with type III SMA. The study reported an improvement in HRQoL over a 14-month period of Nusinersen treatment (53).

Nevertheless, it is important to note that SMA therapies are associated with high costs and uncertain outcomes, as observed in this study and others. Consequently, the Saudi Ministry of Health has engaged in outcome-based agreements with certain therapy manufacturers and implemented programs for SMA patients, which include specific eligibility criteria for accessing these therapies. However, critics of these agreements raise concerns regarding prolonged negotiation periods between manufacturers and payers, which delays access to therapies, strict eligibility criteria that may restrict access for patients who could potentially benefit, as well as potential information bias in assessing various outcomes, data documentation, and governance issues (54, 55). Additionally, the compatibility of these financial agreements with existing laws and regulations in Saudi Arabia has been questioned (56). Therefore, it is crucial to invest in health technology infrastructure, promote increased information sharing and transparency between payers and manufacturers, and reform governance and procurement practices. These steps are necessary to accommodate the rapid pace of innovation witnessed in the field of orphan drugs for rare and ultra-rare diseases (57).

Finally, the impact of premarital screening and newborn screening, along with patient support programs and caregiver training and education, should be examined to effectively address SMA in Saudi Arabia. Creating effective patient support programs that address the identified needs of the patients' and their caregivers in this study are instrumental in providing comprehensive care and enhancing patients' and their families' quality of life. Equally important is caregiver training and education, equipping caregivers with the necessary knowledge and skills to deliver optimal care and effectively navigate the challenges associated with SMA. By implementing these measures improved outcomes for individuals with SMA can be achieved in Saudi Arabia.

## 5 Limitations

The present study has several limitations that should be considered when interpreting the findings. First, due to its cross-sectional design, the establishment of causal relationships is not possible. Additionally, the utilization of snowball sampling introduces information bias and limits the generalizability of the findings. Therefore, future studies should implement multi-stage random probability sampling method to improve both the internal and external validity of the findings. Moreover, the reliance on proxy respondents for interviews increases the risk of information bias, as highlighted in previous studies (58). Furthermore, the potential presence of interviewer bias cannot be ruled out, which may have further contributed to information bias. It should be noted that certain questionnaire items, particularly those pertaining to the quality of sleep among caregivers, exhibited a high non-response rate. Consequently, comparisons of sleep quality across caregivers of SMA patients are constrained by the significant number of unanswered questions, which hinders comprehensive analysis. Moreover, the non-response rate across multiple questionnaire items introduces additional information bias. The presence of acquiescence bias cannot be discounted as well. Lastly, it is important to acknowledge that the assessment of quality of life was conducted using a generic scale instead of a disease-specific scale, which may limit the precision of the results (59).

## 6 Conclusions

Our study indicates that the characteristics of SMA and its subtypes in Saudi Arabia are comparable to those observed in other countries. SMA type I remains the most severe variant, warranting increased attention from healthcare providers and policymakers. Fortunately, the emotional burden on caregivers remains minimal, largely due to the cultural norms established in the country. On the other hand, financial expenditures, while significant, do not correlate with the severity of the disease due to the variable rates of survival. The findings of this study underscore the pressing need to improve societal awareness regarding SMA and its catastrophic consequences, particularly in light of the high rates of consanguineous marriages in Saudi Arabia. To address this issue, the implementation of public awareness campaigns, premarital screening, and newborn screening programs is strongly recommended (60–62). Additionally, publishing the treatment outcomes of SMA patients enrolled in different outcome-based payment programs (OBP) is vital for evaluating the true value of these expensive therapies. Moreover, it is imperative to design and offer various patient-support programs to address the specific needs of both patients and caregivers (63). Future studies with larger sample sizes and more robust analyses should be conducted to examine the direct medical costs and socioeconomic burden of SMA. This research will provide policymakers with valuable insights to develop preventative policies aimed at reducing the incidence of the disease, such as premarital screening.

## Data availability statement

The original contributions presented in the study are included in the article/supplementary material, further inquiries can be directed to the corresponding author.

## Ethics statement

The studies involving humans were approved by Institutional Review Board (IRB) of King Saud University Medical City (Approval of Research Project No. E-22-6955), located in Riyadh, Saudi Arabia. The studies were conducted in accordance with the local legislation and institutional requirements. Written informed consent for participation in this study was provided by the participants' legal guardians/next of kin.

## Author contributions

KA: Data curation, Investigation, Project administration, Writing – original draft. MAls: Data curation, Project administration, Resources, Writing – review & editing. KA-E: Data curation, Methodology, Project administration, Writing – review & editing. AB: Methodology, Project administration, Resources, Supervision, Writing – review & editing. AM: Methodology, Resources, Writing – review & editing. AA: Resources, Supervision, Writing – review & editing. HA-H: Resources, Supervision, Writing – review & editing. SA: Resources, Supervision, Writing – review & editing. FA: Conceptualization, Methodology, Resources, Writing – review & editing. M-HT: Conceptualization, Methodology, Writing – review & editing. NA: Methodology, Project administration, Writing – review & editing. MAlw: Data curation, Methodology, Writing – review & editing. YAs: Project administration, Supervision, Writing – review & editing. YAl: Conceptualization, Formal analysis, Funding acquisition, Investigation, Project administration, Supervision, Writing – review & editing.
